# Defective IL-23/IL-17 Axis Protects p47phox−/− Mice from Colon Cancer

**DOI:** 10.3389/fimmu.2017.00044

**Published:** 2017-01-27

**Authors:** Cornelia Richter, Martina Herrero San Juan, Benno Weigmann, Dominik Bergis, Katrin Dauber, Michael H. Muders, Gustavo B. Baretton, Josef Martin Pfeilschifter, Halvard Bonig, Sebastian Brenner, Heinfried H. Radeke

**Affiliations:** ^1^Department of Pediatrics, University Clinic ‘Carl Gustav Carus’ Dresden, Dresden, Germany; ^2^pharmazentrum frankfurt/ZAFES, Goethe University, Frankfurt, Germany; ^3^I. Medical Clinic, University of Erlangen-Nuremberg, Erlangen, Germany; ^4^Department of Internal Medicine 1, Goethe University, Frankfurt, Germany; ^5^Bristol-Myers Squibb GmbH & Co. KGaA, Munich, Germany; ^6^Department of Pathology, University Clinic ‘Carl Gustav Carus’ Dresden, Dresden, Germany; ^7^German Red Cross Blood Service, Institute for Transfusion Medicine and Immunohematology, Goethe University, Frankfurt, Germany; ^8^Department of Medicine/Hematology, University of Washington, Seattle, WA, USA

**Keywords:** p47phox, DSS/AOM, IL-23, IL-17, chronic colitis, colon cancer, IL23p19 knockout mouse

## Abstract

In the colon, a sophisticated balance between immune reaction and tolerance is absolutely required. Dysfunction may lead to pathologic phenotypes ranging from chronic inflammatory processes to cancer development. Two prominent modulators of colon inflammation are represented by the closely related cytokines interleukin (IL)-12 and IL-23, which initiate adaptive Th1 and Th17 immune responses, respectively. In this study, we investigated the impact of the NADPH oxidase protein p47phox, which negatively regulates IL-12 in dendritic cells, on colon cancer development in a colitis-associated colon cancer model. Initially, we found that IL-12−/− mice developed less severe colitis but are highly susceptible to colon cancer. By contrast, p47phox−/− mice showed lower tumor scores and fewer high grade tumors than wild-type (WT) littermates. Treatment with toll-like receptor 9 ligand CpG2216 significantly enhanced colitis in p47phox−/− mice, whereas tumor growth was simultaneously reduced. In tumor tissue of p47phox−/− mice, the IL-23/IL-17 axis was crucially hampered. IL-23p19 protein expression in tumor tissue correlated with tumor stage. Reconstitution of WT mice with IL-23p19−/− bone marrow protected these mice from colon cancer, whereas transplantation of WT hematopoiesis into IL-23p19−/− mice increased the susceptibility to tumor growth. Our study strengthens the divergent role of IL-12 and IL-23 in colon cancer development. With the characterization of p47phox as a novel modulator of both cytokines our investigation introduces a promising new target for antitumor strategies.

## Introduction

Maintenance of a subtle balance of immune responses in the colon is required to prevent excessive reactions to commensal microbes while at the same time providing effective recognition and removal mechanisms for invading pathogens. This comprehensive task is fulfilled by colon-resident innate immune cells, such as macrophages and dendritic cells (1–4). Unbalanced immune responses may result in autoimmune reactions (excessive responsiveness) or as sequelae of chronic inflammation to tumor initiation (excessive tolerance) on either end of the spectrum. Initiation of a specific adaptive immune response is governed by secretion of polarizing cytokines by activated antigen-presenting cells. The polarizing interleukin (IL)-12 family members IL-12 and IL-23 share the common subunit p40, which is linked to the subunit p35 to form IL-12 or to the subunit p19 to form IL-23 (5, 6). In recent years, an intense debate about both cytokines has arisen with respect to their apparently opposing roles during tumor development. Whereas IL-12 initiates tumor rejection *via* polarization of IFNγ-producing Th1 cells, activation of natural killer, and cytotoxic T cells (7–9), IL-23 promotes tumor progression. IL-23 induces IL-17 and IFNγ secretion of innate lymphocytes and T helper cells in the colon (10, 11). It has been demonstrated that IL-23 is highly upregulated in human tumor tissue from different organs (12). In human colorectal cancer, the expression of IL-23, its receptor (IL-23R), and IL-17 correlates with a poor prognosis (13–15). Human data have been confirmed in tumor models of mice (12, 16, 17). In a very recent publication, Teng and colleagues showed that in an equilibrium phase, characterized by residual, not-eliminated tumor cells, the activation and regulation of IL-12 and IL-23 seem to be important for the progression of malignant tumors (18). In this situation, the balance between IL-12 and IL-23 in the tumor microenvironment is regulated by the transcription factor STAT3 and possibly sphingosine-1-phosphate (19, 20).

A shift of the IL-12/IL-23 balance toward IL-12 might represent an effective antitumor therapy. In murine dendritic cells, we demonstrated that the toll-like receptor (TLR) 9-induced IL-12/Th1 axis is regulated by p47phox, a protein of the NADPH oxidase (21). With the following study, we investigated whether expression of p47phox influences inflammation-dependent tumor growth in mice. For this purpose, we established a colitis-dependent colorectal cancer model (22) combined with the application of the TLR9 ligand CpG. Monitoring the progress of colitis and tumor growth with a mini-endoscope developed for mouse colonoscopy (23), our data clearly support the pivotal roles of IL-12 and IL-23 and the modulation by p47phox in colon cancer.

## Animals and Methods

### Mice

Wild-type (WT) C57BL/6 and IL-12p35−/− mice were obtained from Jackson Laboratory (24). Initial breeding pairs of p47phox−/− transgenic mice also from Jackson Laboratory were kindly provided by R. Brandes and subsequently backcrossed to the C57BL/6 background for 10 generations (25). IL-23p19−/− mice were kindly provided by N. Ghilardi (Genentech) (26). The animals were bred and fed under pathogen-free conditions in secluded scantainers. All animal experiments were approved by the Institutional Animal Care and Use Committee of Goethe University Medical Faculty and the Animal Protection Agency of the Federal State of Hessen.

### Experimental Model of Colitis and Colon Cancer

The inflammation-induced colon cancer mouse model as introduced by Tanaka et al. was adapted to the mouse strains used in this study (22). Specifically, mice received a single intraperitoneal injection (10 mg/kg body weight) of the cancerogenic reagent azoxymethane (AOM, SigmaAldrich). Chronic colitis was induced by three cycles of 1.5% dextrane sodium sulfate (DSS, MP Biomedicals) in the drinking water for 1 week and normal drinking water for the consecutive 2 weeks. Mice received i.p. injections of TLR9 ligand ODN CpG2216 (25 μg/mouse, Invivogen) or phosphate-buffered saline (PBS) once per week. Every day, mice were monitored for their general condition, weight, feces, and bleeding. A detailed scoring protocol is shown in Table 1.

**Table 1:** **Scoring system for inflammation (colitis)**.

Parameter	Score			
	0	1	2	3
Thickening of the colon	Transparent	Moderate	Marked	Non-transparent
Changes of the vascular pattern	Normal	Moderate	Marked	Bleeding
Fibrin visible	None	Little	Marked	Extreme
Granularity of the mucosal surface	None	Moderate	Marked	Extreme
Stool consistency	Normal + solid	Still shaped	Unshaped	Spread

### Endoscopic Procedure and Sample Collection

At the end of the second (week 5) and third (week 8) cycles of DSS, mice were anesthetized and examined endoscopically with respect to inflammation and tumor development in the colon using the coloview system (23). While the colonoscopy at week 5 was performed to control for disease progression, during the second colonoscopy serial high resolution micrographs covering the entire colon were recorded, and severity of inflammation and tumor growth was numerically scored in a blinded fashion with a scoring system described previously and represented in Tables 1 and 2 (23, 27). Finally, mice were sacrificed, the colon was dissected and washed with PBS, and samples for RNA, protein, and histology were taken. Discrimination of tumor and inflamed tissue based on the recorded colonoscopy pictures. Samples for RNA isolation were stored in RNA later (Qiagen) at 4°C until their use. Tissue samples for protein isolation were immediately frozen in liquid nitrogen and stored at −80°C. Histology samples were frozen and stored at −80°C.

**Table 2:** **Scoring system for tumor growth**.

Number of tumors	Score	Size (average diameter)	Score
0	0	0	0
1–2	1	Just detectable	1
3–5	2	1/8	2
6–8	3	1/4	3
9–12	4	1/2	4
≥13	5	≥1/2	5

### Allogeneic Bone Marrow Transplantation

Bone marrow cells were isolated from femurs and tibiae of donor WT and IL-23p19−/− mice by flushing the bones with PBS containing 0.5% BSA (Sigma Aldrich). Bone marrow cells were counted and the hematological profile was determined (Hemavet 950FS; Drew Scientific). Groups of WT and IL-23p19−/− mice (aged 10 weeks) were irradiated (9.5 Gy) and within 1 h 1 × 10^6^ bone marrow cells in 200 µl PBS were injected intravenously into the tail vein. The transplantation scheme (donor and recipient) is shown in Table 3. Transplanted mice received antibiotic treatment (0.025% Baytril, Bayer HealthCare) in the drinking water and jelly food for 10 days after transplantation and were monitored at least daily. Mice with >15% weight loss received systemic fluid supplementation with 300 µl warmed Sterofundin G (B Braun Melsungen AG) given as i.p. bolus. Following confirmation of a full immune reconstitution, the DSS/AOM experiment was initiated 16 weeks after transplantation.

**Table 3:** **Transplantation scheme**.

Recipient	Donor	
	Wild-type (WT)	Interleukin (IL)-23p19−/−
WT	✓	✓
IL-23p19−/−	✓	✓

### RNA Isolation and Quantitative Real-time PCR

RNA from colon tissue samples was isolated using the RNeasy Mini Kit (Qiagen, Hilden, Germany) and the concentration of RNA was determined photometrically. Synthesis of cDNA was performed with the High Capacity cDNA Reverse Transcription Kit (Applied Biosystems) using random hexamers and 0.5 µg of total RNA according to the manufacturer’s instructions. Expression of mRNA was analyzed using specific TaqMan^®^ Gene Expression Assays containing 20× pre-formulated primers and 5′FAM-labeled probes (Table 4; Applied Biosystems). Quantitative real-time PCRs were performed in triplicates with 5 µl AbsoluteFast QPCR LowROX Master Mix (ABgene), 3.5 µl H_2_O, 0.5 µl of 20× assay mix, and 1 µl cDNA with the following cycling program: 95°C for 5 min (1×) then successively 95°C for 3 s and 60°C for 30 s (40×) in the 7500 Fast Real-Time PCR System (Invitrogen). For each gene analyzed, a normalized ratio was calculated using 18s RNA as a reference gene and cDNA from non-treated mice as control to compensate for inter-run differences.

**Table 4:** **Target genes and their corresponding gene expression assays**.

Gene of interest	TaqMan^®^ gene expression assay ID number
Interleukin (IL)-23p19	Mm00518984_m1
IL-12p35	Mm00434165_m1
IL-12/23p40	Mm01288993_m1
IL-17A	Mm00439619_m1
18s	Hs03003631_g1

### Flow Cytometry

Isolation of mononuclear cells from colon tumor tissue was performed as described previously (28). Single cell suspension was washed twice in PBS with 0.5% BSA and 2 mM EDTA and stained with antibodies for CD45-PE-Cy7, CD11b-V500, CD11c-PerCP, IL-12p35-PE, and IL-23p19-APC (BD Biosciences). Flow cytometric analyses were conducted using an LSR II flow cytometer (BD Biosciences). Data were evaluated using FlowJo software (Version 7.6.5; FlowJo).

### Immunohistochemistry

Frozen tissue samples were fixed in 3.7% formaldehyde (FA) overnight at 4°C, transferred into 1% FA until they were embedded in paraffin. Paraffin samples were cut into 5 µm sections and stained with hematoxylin and eosin. Tumor grades in histological sections were classified into aberrant crypt foci (ACF), adenoma (Ad), and mucosal carcinoma. Detection of IL-23 in the tumor tissue was performed with an anti-IL-23p19 antibody (rabbit polyclonal, Abcam). Proliferating cells were detected *via* Ki-67 staining with a rat monoclonal antibody (Dako). Secondary anti-rabbit and anti-rat (Vector Laboratories, Inc.) antibodies coupled to biotin were added and sections were developed *via* the streptavidin–HRP VECTASTAIN^®^ ABC Kit (Vector Laboratories, Inc.). Finally, color development was performed with 3-amino-9-ethylcarbazole (AEC; Dako). All sections were counterstained with Harris hematoxylin solution (Sigma Aldrich).

### Statistics

Unless otherwise indicated, experiments were performed at least three times with three to six animals per group. Data are presented as the mean ± SEM. Statistical significance was tested with Mann–Whitney *U*-test or conditional Wilcoxon *U*-test with *P*-values corrected by Holm for multiple testing.

## Results

### IL-12p35−/− Mice Exhibit Reduced Colitis but Increased Susceptibility to Colon Cancer

**Figure 1:**
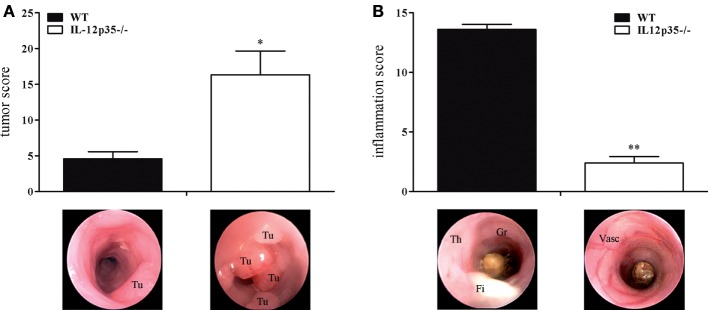
**Tumor and inflammation score of interleukin (IL)-12p35−/− and wild-type (WT) mice**. IL-12p35−/− and WT mice were investigated in the DSS/AOM model. **(A)** Tumor score and **(B)** inflammation score of IL-12p35−/− and WT mice were determined by colonoscopic examination. Representative endoscopic pictures are shown (Tu, tumor; Th, thickening of the colon; Gr, granularity of the mucosal surface; Fi, fibrin; Vasc, vascularization). Results are mean ± SEM of at least six mice per group. Statistical significance is indicated (*P < 0.05; **P < 0.01).

Although they share a common subunit, IL-12 and IL-23 have divergent immunological roles regarding chronic inflammation and cancerogenesis. The protective role of IL-12 in cancerogenesis has been demonstrated in chemically or physically (UV light) induced or spontaneous cancer models (12, 16, 17, 29). To examine the role of IL-12 in colitis-associated colon cancer, we compared IL-12p35−/− mice with WT mice for their susceptibility to cancer in the DSS/AOM model. Compared to WT mice, IL-12p35−/− exhibited a significantly higher tumor score (Figure 1A). By contrast, IL-12p35−/− mice developed significantly milder DSS colitis compared to WT mice (Figure 1B). These data strengthen the protective function of IL-12 in inflammation-dependent colon cancer.

### Increased Inflammation but Decreased Tumor Growth in p47phox−/− Mice

**Figure 2:**
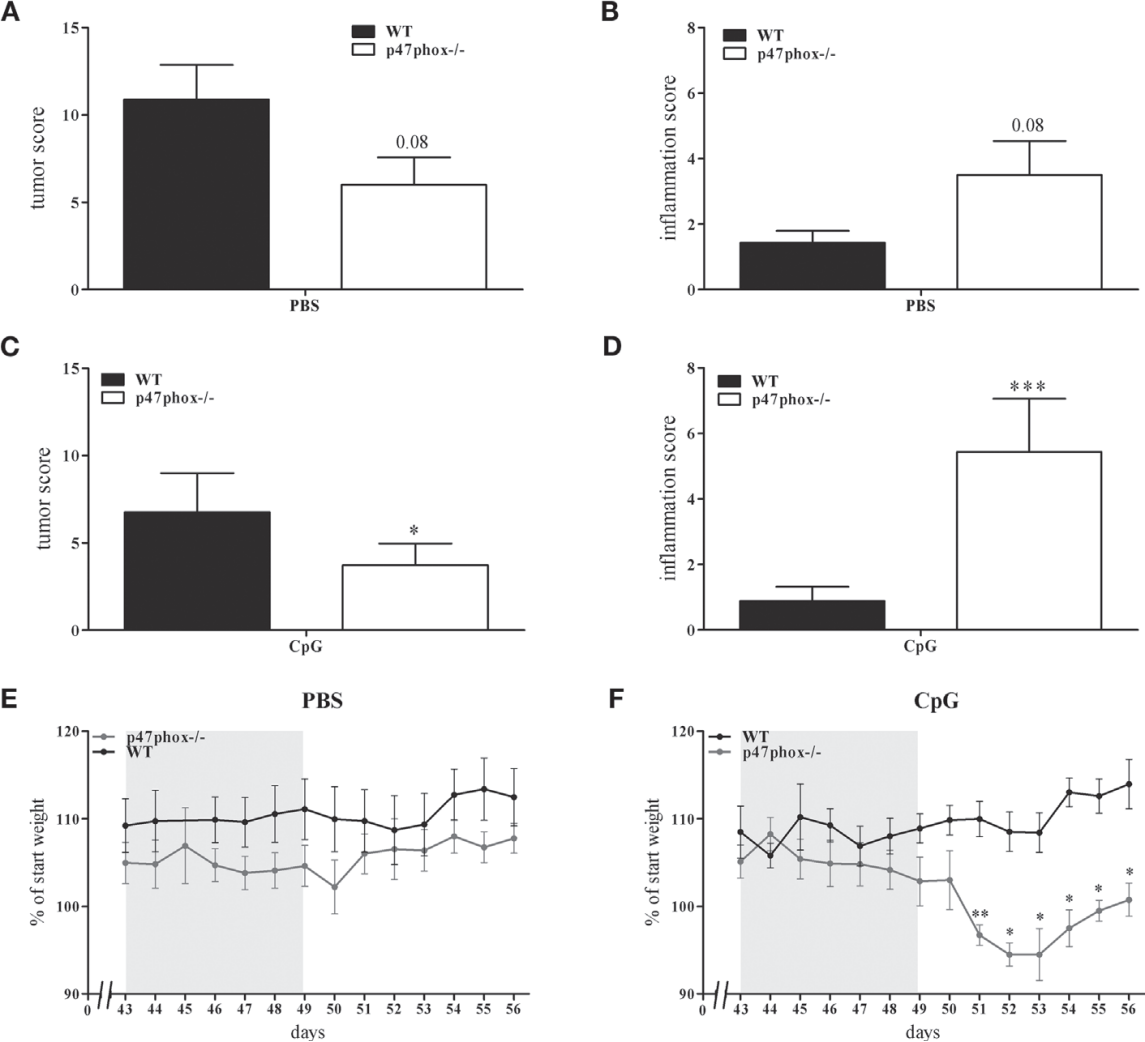
**Tumor and inflammation score of p47phox−/− and wild-type (WT) mice treated with or w/o toll-like receptor 9 ligand CpG**. **(A)** Tumor score and **(B)** inflammation score of p47phox−/− and WT mice, determined by colonoscopic examination. **(C)** Tumor score and **(D)** inflammation score of p47phox−/− and WT mice, weekly treated with 25 µg CpG2216. **(E,F)** Weight development of mice treated w/o **(E)** and with CpG **(F)** during and after the third DSS cycle as percentage of starting weight. The DSS cycle is marked in gray. **(A–F)** Results are mean ± SEM of three independent experiments with at least seven mice per group. Statistical significance is indicated (*P < 0.05; **P < 0.01; ***P < 0.001).

In a previous study, we showed an increased, TLR9-induced IL-12 response in p47phox−/− mice (21). These mice are characterized by a defective NADPH oxidase (nox2) system due to lack of p47phox which results in decreased reactive oxygen species and increased inflammation. In order to prove whether the augmented IL-12/Th1 axis may influence tumor development, we investigated p47phox−/− and WT mice in the DSS/AOM tumor model. During endoscopic examination of mice, we detected a decreased tumor score in p47phox−/− mice (6.0 ± 1.58, *n* = 8; Figure 2A) compared to WT mice (9.43 ± 2.26, *n* = 7; Figure 2A). By contrast, the inflammation score was increased in p47phox−/− mice (3.5 ± 1.04, *n* = 8; Figure 2B) in comparison to WT mice (1.43 ± 0.37, *n* = 7; Figure 2B). Investigating the influence of TLR9-dependent activation of immune cells during the tumor development, mice were treated with CpG ODN 2216 (CpG). Application of CpG suppressed the tumor development in WT and p47phox−/− mice compared to the PBS group (Figure 2C vs. Figure 2A). Nevertheless, tumor scores were significantly lower in CpG-treated p47phox−/− mice (3.71 ± 1.25, *n* = 7; Figure 2C) compared to WT (6.75 ± 2.24, *n* = 8; Figure 2C). However, in CpG-treated p47phox−/− mice, we detected a significant enhanced inflammation score compared to WT mice (p47phox−/−: 5.38 ± 1.41, *n* = 7; WT: 0.88 ± 0.44, *n* = 8; Figure 2D). Weight loss, a characteristic albeit unspecific symptom of colitis, was monitored in detail during the third DSS cycle (chronic inflammation). A few days after the final DSS application, we quantified the weight loss. Between non-treated (PBS) WT and p47phox−/− animals no weight difference was detected (Figure 2E). By contrast, we observed a higher weight loss in CpG-treated p47phox−/− mice compared to WT mice (Figure 2F).

### Tumor-Promoting IL-23/IL-17 Axis Is Impaired in p47phox−/− Mice

**Figure 3:**
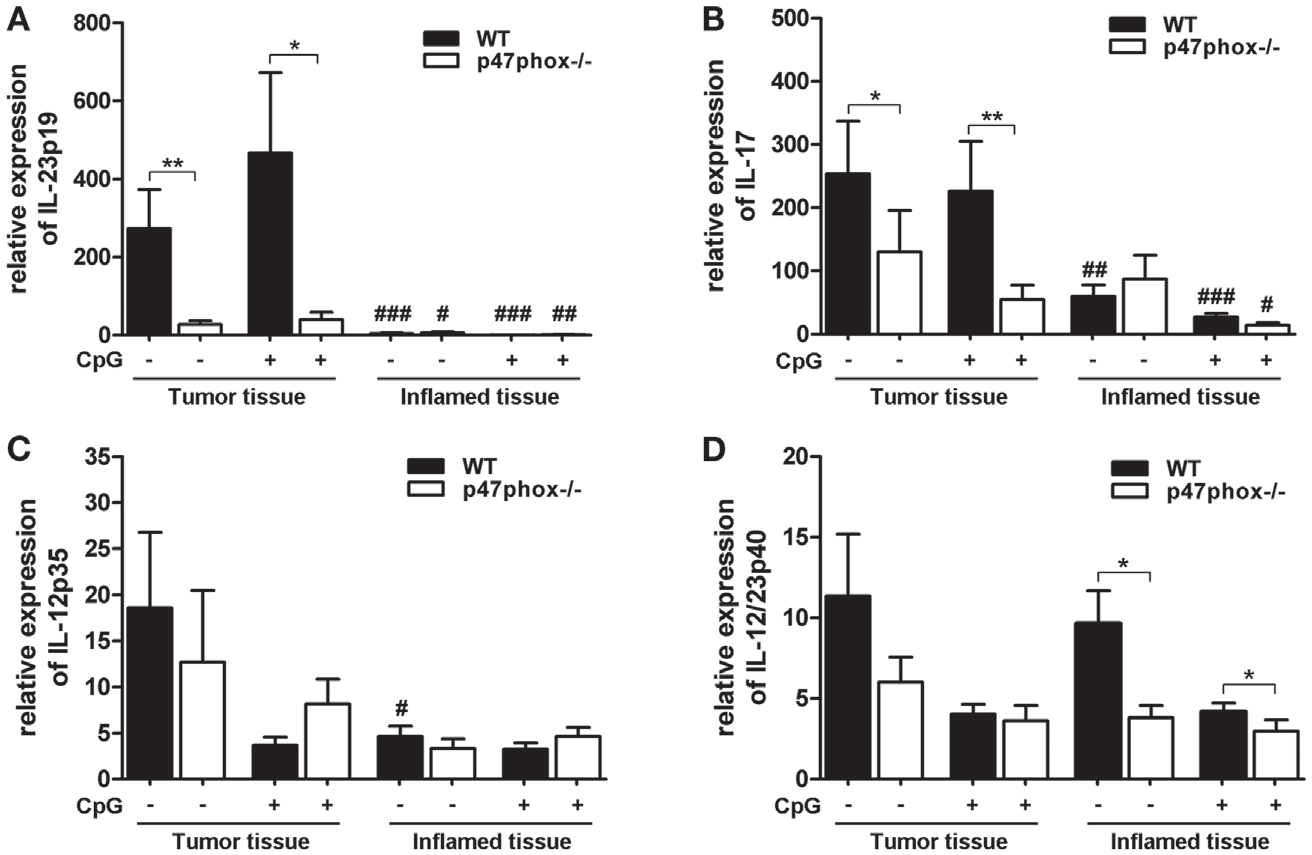
**Expression of interleukin (IL)-12, IL-23, and IL-17 mRNA in tumor and inflamed tissue of p47phox−/− and wild-type (WT) mice**. **(A)** Expression of IL-23p19, **(B)** IL-17, **(C)** IL-12p35, and **(D)** IL-12/23p40 mRNA in tumor and inflamed tissue from p47phox−/− and WT mice treated with or w/o CpG was determined by quantitative real-time PCR. Results are mean ± SEM and representative of three independent experiments with *n* = 12–22 samples. General differences between p47phox−/− and WT mice were tested for statistical significance (*P < 0.05; **P < 0.01). Differences between tumor and inflamed tissue from p47phox−/− and WT mice, respectively, were tested for statistical significance (^#^*P* < 0.05; ^##^*P* < 0.01; and ^###^*P* < 0.001).

Based on endoscopic analyses, we wanted to know which inflammatory and tumor-promoting cytokines are involved. We analyzed the mRNA of tumor and inflamed colon tissue from WT and p47phox−/− mice treated with or w/o CpG. In both WT and p47phox−/− mice, we detected a significantly increased IL-23p19 mRNA expression in tumor tissue compared to inflamed tissue (Figure 3A). Moreover, tumor samples from WT mice showed significantly higher expression of IL-23p19 mRNA when compared with their p47phox−/− littermates (Figure 3A). IL-23 drives the cytokine IL-17, which is also associated with tumorigenesis. We detected an increased IL-17 mRNA expression in tumor tissue compared to inflamed tissue (Figure 3B). As seen for IL-23p19, we also found significantly higher IL-17 mRNA expression in WT tumor tissue compared to p47phox−/− mice. Treatment with CpG did not significantly influence IL-23p19 or IL-17 mRNA expression (Figures 3A,B).

Based on our previously published data, that CpG-induced IL-12 is significantly increased in p47phox−/− dendritic cells, we investigated the expression of IL-12 during colitis-associated colon cancer. In contrast to IL-23p19, we did not detect significant differences for IL-12p35 mRNA expression between WT and p47phox−/− mice (Figure 3C). Interestingly, compared to inflamed tissue, IL-12p35 mRNA is significantly increased in tumor tissue of WT mice that were not treated with CpG (Figure 3C).

The common IL-12 and IL-23 subunit p40 exhibited no difference in the mRNA expression level between tumor and inflamed tissue (Figure 3D). Nevertheless, in inflamed tissue p47phox−/− mice expressed significantly less IL-12p40 mRNA compared to WT mice (Figure 3D). In summary, our data revealed a significantly impaired IL-23/IL-17 axis in tumor tissue of p47phox−/− mice.

### Lower Tumor Grades in p47phox−/− Mice and the Correlation with IL-23 Protein Expression

**Figure 4:**
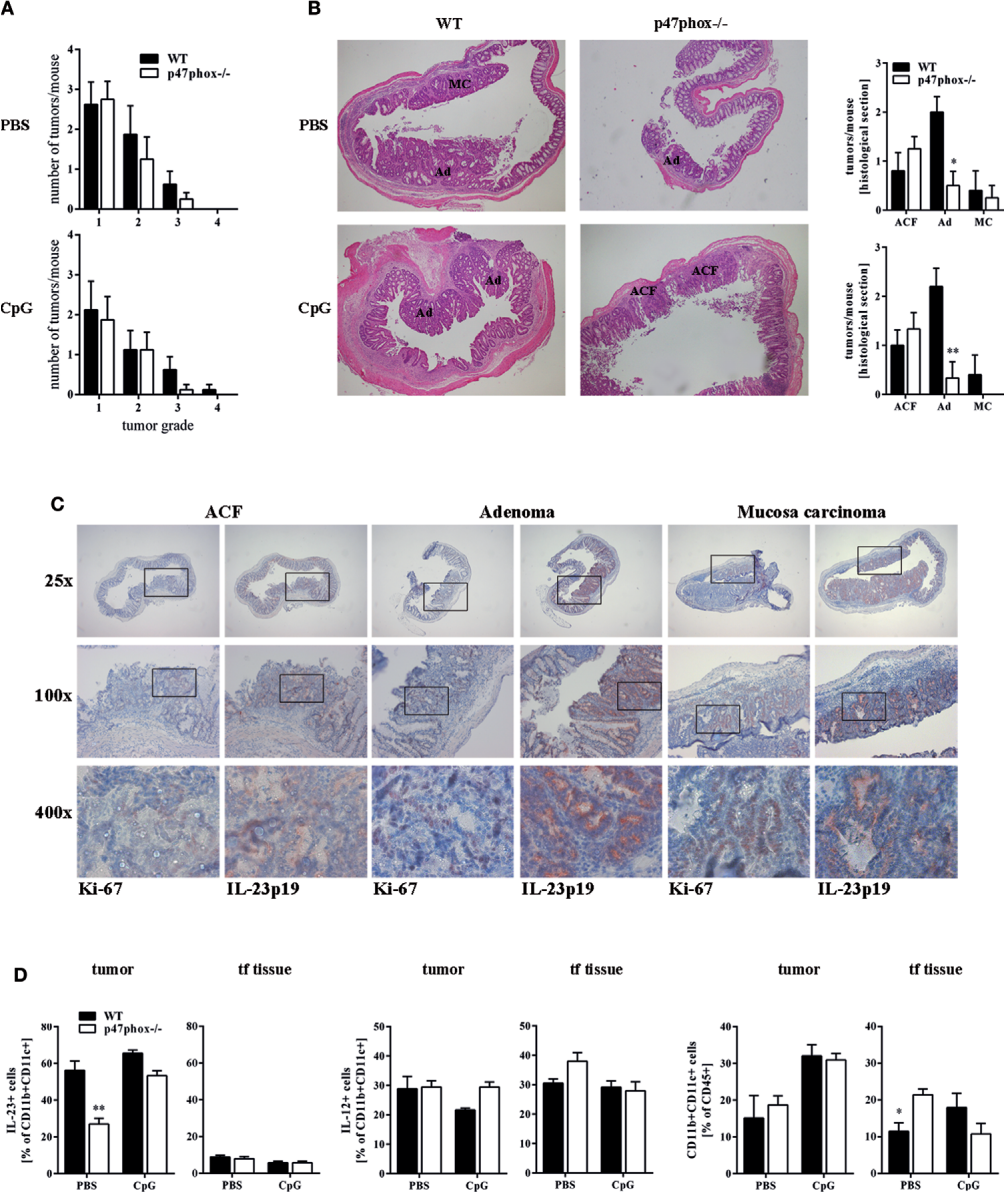
**Histological analyses of tumor tissue**. **(A)** Numbers of tumors per tumor size calculated from seven mice per group. **(B)** Colon tissue sections of p47phox−/− and wild-type mice treated with or w/o CpG were stained with hematoxylin and eosin. Grades are indicated as aberrant crypt foci, adenoma, and mucosa carcinoma (MC). One representative section of three to five is shown. Statistical analysis of tumors in histological sections was performed. Results are mean ± SEM of three to five histological samples per group **(C)** Colon tissue sections were stained for interleukin (IL)-23, Ki-67, and counterstained for hematoxylin. For each stage, one representative section of three to five is shown. **(D)** Flow cytometry analysis of IL-23 and IL-12 in myeloid cells from tumor and tumor-free tissue was performed. Results are mean ± SEM of five to seven mice per group. Statistical significance is indicated (*P < 0.05; **P < 0.01).

Our endoscopic evaluation showed that p47phox−/− mice generally exhibit lower tumor scores. Compiling and sorting all tumors prepared from colons of WT and p47phox−/− mice related to their size, we observed that p47phox−/− mice develop equal numbers of grade 1 and 2 tumors, but fewer grade 3 and 4 tumors than WT mice (Figure 4A). In colon sections, stained with H&E, we detected different tumor grades. In contrast to WT mice, p47phox−/− mice develop some more ACF, but significantly fewer Ad and specifically after CpG treatment no MC (Figure 4B). Recently, it has been shown that IL-23p19 mRNA expression is strongly upregulated in human and mouse colorectal cancer (13, 14, 16). To prove whether IL-23 expression correlates with the tumor grade, we stained colon sections from WT mice for IL-23p19 protein. We could hardly detect IL-23p19 in ACF (Figure 4C). By contrast, IL-23 expression increased with the tumor grade (Figure 4C). In detail, Ad and MC tissue exhibited dramatically increased IL-23p19 protein (Figure 4C). In parallel, we stained the sections for Ki-67 to detect the proliferating cells within the different tumor stages (Figure 4C). To determine the cell source of IL-23 in tumor tissue, we isolated mononuclear cells from tumor and tumor-free tissue and analyzed them for the cytokines IL-23 and IL-12 (Figure 4D). We detected in CD11b+CD11c+ myeloid cells, isolated from colon tumors high level of IL-23 (Figure 4D, left). In contrast to WT cells, p47phox−/− myeloid cells produce less IL-23 in tumor tissue. In tumor-free tissue, only a few myeloid cells secrete IL-23 independent of the genetic background. In contrast to IL-23, IL-12 was slightly increased in p47phox−/− myeloid cells, but no significant difference could be observed (Figure 4D, middle). Furthermore, we measured equal numbers of myeloid cells in tumor tissue between WT and p47phox−/−, whereas in tumor-free tissue from p47phox−/− PBS-treated mice, the percentage of myeloid cells was significantly increased (Figure 4D, right).

### Allogeneic Transplantation of IL-23p19−/− Bone Marrow Cells Protected WT Mice against Colon Cancer in a TLR9-Dependent Manner

In the tumor microenvironment, tumor-infiltrating CD11b+ or F4/80+ myeloid cells appear to be the main source of IL-23 (16). In our model, the source of IL-23 in tumor tissue are CD11b+CD11c+ myeloid cells (Figure 4D). Using a lethal irradiation/bone marrow transplantation approach, with isogeneic WT or IL-23p19−/− hosts and WT or IL-23p19−/− donor hematopoietic cells, we sought to address whether bone marrow-derived myeloid cells infiltrate colon tissue and influence TLR9-dependently chronic inflammation and tumor progression in the colon.

**Figure 5:**
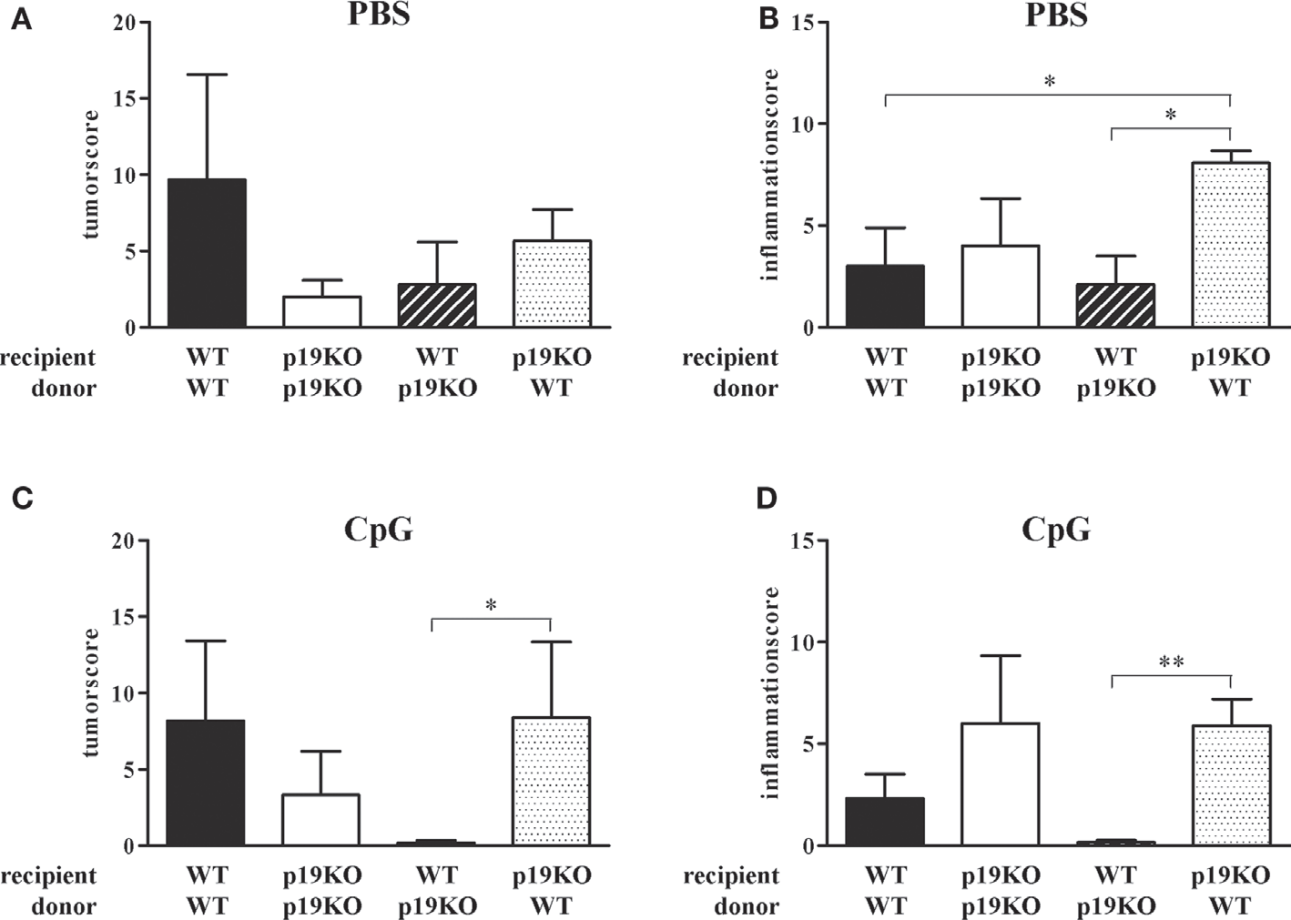
**Tumor and inflammation score of interleukin (IL)-23p19−/− (p19KO) and wild-type (WT) recipient mice**. **(A,B)** Tumor **(A)** and inflammation **(B)** score of p19KO and WT mice, lethally irradiated and transplanted with bone marrow cells from p19KO and WT mice or *vice versa*. **(C,D)** Tumor **(C)** and inflammation **(D)** score of CpG-treated, transplanted mice. Results are mean ± SEM and *n* = 3–6. Statistical significance is indicated (*P < 0.05; **P < 0.01).

As expected from previous publications, IL-23p19−/− bone marrow-transplanted IL-23p19−/− mice showed decreased tumor growth in comparison to WT animals reconstituted with WT cells (Figure 5A, white vs. black bars). Treatment with TLR9 ligand CpG2216 did not influence the tumor score in WT/WT or IL-23p19−/−/IL-23p19−/− mice (Figure 5C). Interestingly, WT mice, transplanted with bone marrow cells from IL-23p19−/− mice have lower tumor scores compared to WT controls (Figure 5A, black-white hatched vs. black bars) and were protected from tumor growth after CpG treatment (Figure 5C, black-white hatched bar), i.e., showed the same phenotype as IL-23p19−/−/IL-23p19−/− mice. Correspondingly, transplantation of WT bone marrow cells into IL-23p19−/− mice resulted in increased tumor growth which became significant when mice were treated with CpG (Figures 5A,C, black dotted bars), indicating that hematopoietic cell-intrinsic effects mediate the promotion of tumor growth in our model. While we observed no differences in the inflammation score between WT and IL-23p19−/− mice (Figure 5B), CpG-treated IL-23p19−/− showed increased inflammation compared to WT mice (Figure 5D). Transplantation of WT bone marrow cells into IL-23p19−/− mice resulted in significantly enhanced inflammation compared to WT/WT (Figures 5B,D, black-white hatched bars). By contrast, WT mice transplanted with IL-23p19−/− bone marrow showed significantly less inflammation compared to IL-23p19−/− mice with WT hematopoiesis (Figure 5D).

In summary, ablation of IL-23p19 specifically in the hematopoietic compartment is sufficient to replicate the anti-neoplastic phenotype of the IL-23p19−/− mice, while surprisingly ablation only in stroma had the opposite effect. Furthermore, modulation of TLR9 in WT mice reconstituted with IL-23p19−/− bone marrow cells strongly enhanced the protection against colon tumors and chronic inflammation.

## Discussion

The cytokines IL-12 and IL-23 share the subunit p40, which heterodimerizes with the subunit p35 to form bioactive IL-12 or with p19, to generate bioactive IL-23 (30). While both pro-inflammatory cytokines are associated with autoimmune diseases (31, 32), the balance between IL-12 and IL-23 seems to be critical for the outgrowth of malignant cancer (18).

In this study, we wanted to ascertain whether the modulation of IL-12 and IL-23 affects carcinogenesis in the colon. In initial experiments, we investigated IL-12p35−/− mice for their susceptibility to colon cancer. In a colitis-associated cancer model (DSS/AOM model), we confirmed the data from others that IL-12 deficiency resulted in increased tumor growth. In a previous publication, we described a negative feedback mechanism of IL-12 by the NADPH oxidase protein p47phox (21). These findings raised the question, whether the modulation of IL-12 by the p47phox influences the tumor development in this colitis-associated cancer model. Indeed, we found a decreased tumor score in p47phox−/− mice compared to WT mice (Figure 2A). By contrast, inflammation in the colon was increased in p47phox−/− mice (Figure 2B). Recently, more severe colitis has been demonstrated in the p47phox mutant strain BQ.*Ncf1m1J/m1J*, which we have investigated for the IL-12 feedback mechanism (21, 33). In our model, repeated cycles of DSS results in chronic colitis which promotes tumor development (22). Interestingly, we observed that inflammation in the colon is uncoupled from tumor growth in p47phox−/− mice. Because the balance of the two closely related cytokines IL-12 and IL-23 is important for the switch from chronic inflammation to tumorigenesis (18, 19), we wanted to know whether this could be an explanation for the divergence between inflammation and tumor growth in p47phox−/− mice. In this context, we investigated the mRNA expression of IL-12 and IL-23 subunits. Whereas we detected high amounts of the IL-23 subunit p19 in tumor tissue of WT mice; in p47phox−/−, we observed significantly lower p19 expression (Figure 3A). In recent years, IL-23 has emerged as an important player in the development of cancer. In various human tumors, IL-23 mRNA is increased and correlates with a bad prognosis (12, 14). Furthermore, the IL-23-driven cytokine IL-17, secreted by T cells of the innate and adaptive immune system, is strongly associated with tumor growth (34–36). In our model, we also found increased IL-17 mRNA expression in tumors of WT mice, but not in p47phox−/− mice (Figure 3B). A p47phox-dependent effect on IL-23/IL-17 axis in colon cancer has not been described so far. Evidence was provided that in human colorectal cancer, NADPH oxidase components p47phox and p67phox and the myeloid marker CD14 correlate with IL-23p19 expression levels (12). Defect in p47phox protein expression results in increased IL-6 and IL-17 expression in the chronic DSS colitis model (33). Further, activation of neutrophils and their infiltration into tumor tissue is known to be dependent on IL-23, which induces the secretion of IL-17 and G-CSF from activated T cells. Maintaining constant numbers of circulating neutrophils in the body, dendritic cells, and macrophages phagocytose apoptotic neutrophils, which in turn inhibit the IL-23 expression of those cells (37). It should be mentioned that IL-23 seems to have antitumorigenic potential. Indeed, it has been demonstrated that systemic treatment or local administration of IL-23 induces antitumor immunity, but in contrast to endogenous IL-23 level only excessive exogenous doses led to an antitumorigenic response (38, 39). Furthermore, in ovarian cancer, it was shown that high intra-tumoral IL-23p19 mRNA levels correlate with a better outcome of the disease (40). Interestingly, the authors figured out that high IL-23p19 mRNA expression only improves the survival in early stages of cancer, whereas in late stages high IL-12p35 mRNA is associated with a better outcome.

In our previous study, we demonstrated TLR9-dependent IL-12/Th1 regulation by p47phox (21). To investigate the effect of TLR9 activation, we treated the mice with TLR9 ligand CpG2216 in the DSS/AOM model. Although CpG treatment reduced tumor scores in both, p47phox−/− and WT mice, the lack of p47phox resulted in a significantly enhanced protection against colon cancer development (Figure 2C). By contrast, CpG treatment caused increased inflammation in p47phox−/− mice (Figure 2D), which confirms previous data from inflammation models (41–43). Moreover, we measured a higher, although not significant IL-12p35 mRNA expression in tumor and inflamed tissue from p47phox−/− mice compared to WT mice (Figure 3C). These data support the hypothesis that p47phox-dependent regulation of TLR9-induced IL-12 might be involved in the modulation of the immune response during tumorigenesis. It was shown that IL-23 is the driving cytokine in intestinal inflammation, whereas IL-12 seems to have a more systemic impact (44). In a recent study, using IL-23p19 deficient mice on a dominant negative TGFβ receptor background, it was demonstrated that colon inflammation is IL-23 dependent, but the driving activity for liver inflammation is limited to the IL-12/Th1 axis (45). Although many studies investigated the impact of IL-12 and IL-23 during intestinal inflammation, the exact interaction mechanism of these cytokines remains undefined.

In our study, we detected that p47phox deficient mice generally have lower tumor scores. Interestingly, compared to WT mice they developed similar numbers of low grade tumors (size 1), but fewer high grade tumors (size 3–4; Figure 4A). On histological level, p47phox−/− mice develop some more ACF, but significantly fewer Ads (Figure 4B). Furthermore, with increasing malignancy (from ACF to MC), we found progressively elevated levels of IL-23p19 protein in colon sections (Figure 4C). Together with the significantly reduced IL-23p19 mRNA in tumor samples of p47phox−/− mice, we hypothesize that a p47phox-dependent mechanism, e.g., IL-12/Th1 regulation, shifts the immune balance toward colorectal cancer.

Tumor-infiltrating myeloid cells were previously postulated to be the prime source of IL-23 (16). We confirmed these data by analysis of IL-23 in myeloid cells from tumor and tumor-free tissue (Figure 4D). To directly assess this, as well as to address a possible adjunct role of stroma-derived IL-23, the colitis-associated colon cancer model was also tested in WT mice stably reconstituted with IL-23p19−/− hematopoiesis and in IL-23p19−/− mice reconstituted with WT hematopoietic cells. IL-23p19−/− to IL-23p19−/− and WT to WT transplantation experiments served as controls, i.e., to address whether lethal irradiation and bone marrow transplantation would affect the course of colitis and tumorigenesis. This was not the case, as either recipient groups responded with the same phenotype as non-transplanted IL-23p19−/− or WT mice, respectively. Mice exclusively deficient for IL-23p19 in their hematopoietic system reproduced the phenotype of IL-23p19−/− complete knock-out mice, including the observation that after CpG, the hematopoietic ablation of IL-23p19 strongly attenuated tumor induction, supporting the paradigm mentioned above that the hematopoietic origin of IL-23 is the decisive enhancer of tumor growth (Figure 5). By contrast, IL-23-p19−/− mice bearing WT hematopoietic cells were characterized by a markedly increased colitis, without any effect on the colon tumor score (Figure 5). This observation suggests differential, cell-context-dependent roles of IL-23 on inflammation and tumorigenicity. Several studies imply a separate intestinal function of IL-23/Th17 and IL-12/Th1 *in vivo*, but a recent study demonstrated that Th17 cells convert into Th1 cells promoted by mucosa-derived IL-12 and IL-23 (46). In contrast to this cooperation between IL-12 and IL-23 during colon inflammation, the divergence of both cytokines is critical for the initiation of colon cancer (18).

In conclusion, our study revealed a novel p47phox-dependent regulation of the IL-23/Th17 axis during colon cancerogenesis. Together with the finding that p47phox regulates TLR9-induced IL-12 expression in myeloid dendritic cells; targeted manipulation of p47phox protein would be a promising strategy to shift the IL-12/IL-23 balance into an antitumorigenic direction.

## Author Contributions

CR, KD, HB, JP, SB, and HR designed the experiments, interpreted the data, and wrote the manuscript. CR, MJ, BW, KD, and HB performed the *in vitro* and *in vivo* studies. CR, MJ, DB, KD, MM, and HR performed data analysis and statistical evaluation. GB, JP, HB, SB, and HR revised the work critically and did the final approval of the manuscript before submission.

## Conflict of Interest Statement

The authors declare that the research was conducted in the absence of any commercial or financial relationships that could be construed as a potential conflict of interest. The reviewers GG-A and CA and handling Editor declared their shared affiliations, and the handling Editor states that the process nevertheless met the standards of a fair and objective review.

## Abbreviations

ACF, aberrant crypt foci; AOM, azoxymethane; DSS, dextrane sodium sulfate; IL, interleukin; i.p., intraperitoneal; ODN, oligodeoxynucleotide; PBS, phosphate-buffered saline; STAT, signal transducers and activators of transcription.
